# Keen on the tenure track job, are you? Know these things, you should

**DOI:** 10.1186/s13059-018-1617-8

**Published:** 2019-01-07

**Authors:** Benjamin F. Voight

**Affiliations:** Department of Systems Pharmacology and Translational Therapeutics and Department of Genetics, University of Pennsylvania, Perelman School of Medicine, 3400 Civic Center Boulevard, Philadelphia, PA 19104 USA

## Abstract

Success along the tenure track requires more than hard work and long hours. Here, the experiences of a recently tenured professor are distilled into a collection of tips to assist others along the path.

About six months into starting my tenure track position, I distinctly recall thinking: what job have I *actually* accepted? Academia was the singular place where I could imagine having the freedom to explore all of my scientific interests. It was a combination of hard work, support from mentors, tenacity, networking, preparedness, and good luck that helped vault me into this career—several points of which were articulated in a previous *Comment* in this journal about landing a tenure track job [1]. Having been in the position for a few years when that article appeared, it occurred to me that there might be something helpful to be added regarding what comes next—because, once I had the job, I quickly realized that there was a lot more to it than I ever imagined.

Thinking back over my sojourn through the early-career stage, I still have much to learn. While I continue to figure out how to do this job the very best that I can, now as a tenured professor, I am helping my first cadre of graduate students along their next career steps and am asked to mentor junior faculty through the tenure process. To help them prepare, I’ve increasingly felt the need to inventory my *learning moments* into discrete pieces of advice. My experiences are probably common to young faculty, and, for the sake of brevity, I can only enumerate a subset of them here. It is also true that each person’s experience comes with its own unique set of dynamics and nuance. That said, I hope that these thoughts will resonate with those embarking upon the academic path as a new investigator, to help them better prepare for and accept the realities of the tenure track job that I have come to appreciate.

## Reality of the job—Success is the surprising result

First comes the hard medicine—most ‘shots’ you take will miss and your colleagues will appear to walk on water. Your papers will frequently be rejected. Grant funding is notoriously hard to land and your grant proposals will often be triaged. Trainees will turn down offers to join your lab because everyone is vying for the same rock-star students and post-docs. Your institution will have been recruiting great people for years, and *all of you* are trying to make it. Internally, this means that everyone is competing for the same opportunities that you are. Do not take these disappointments personally. Realize that every new faculty member faces many of these same challenges.

For me, this was one of the hardest realities to accept. It helps me to *avoid lingering* on outcomes that are less than ideal. With persistent focus on progressing towards the horizon, rather than keeping an iron grip on the frustration *du jour*, I can keep the wheels of the lab turning. I also find that having like-minded colleagues at a similar career stage, with whom experiences can be communally exchanged, is quite helpful for maintaining my sanity (more on this later).

It’s crucial to learn whatever you can from specific failures—but what is *really* important is *how you choose to respond to repeated failures*. Chin up, keep at it, chip away, pivot. Work hard at each attempt, make thoughtful choices that sit well with you and execute. Eventually, a few things will break your way. *Cherish the breaks that go your way*, including those that are not scientific (i.e., personal or life goals). These successes will keep you going.

## Reality of the job—Establish your lab’s ethos

The culture and spirit of your lab group is ultimately set by *you*. This ethos plays out both explicitly and implicitly—how, where, and when you interact with students, the scientific demands you place on them (and their levels of stress), the way you set and communicate lab policies, how much you choose to socialize with your group, appropriate lab behavior, ... the list goes on. You must decide and establish core principles and make them clear. These choices come with trade-offs that you should be willing to accept. Each lab environment is different. It is crucial for students to know what expectations you have set so that they can select wisely from amongst prospective thesis labs. But establishing core principles also allows *you* to select the students that you expect will thrive in your lab’s environment.

One challenge with this paradigm is that the environment does change over time. This is especially true as people come and go. While strong personalities might join the group and change the dynamics, ultimately you can choose whether those shifts are consistent with your vision. Sometimes your requirements for the lab change as scientific objectives evolve. Remember—your group ultimately responds to the directions that you give—or choose not to give.

## Reality of the job—Build an appropriate social network

I still remember that moment when, shortly after starting, I was *all alone* in my office and lab. That solitary feeling can require some adjustment. Unlike your previous position, there might not be someone nearby you can grab a coffee with to troubleshoot an experiment, or high-five when a paper is finally accepted. Moreover, your new job comes with a completely new set of social norms, personalities, and array of interactions amongst faculty and administrative support staff within and beyond your department.

To manage this interpersonal landscape, you will need to develop local social and scientific communities. Although it involved a bit of additional service, I found that active engagement in the faculty communities of both of my affiliated departments was useful for social support. I also developed friendships with like-minded faculty who are at similar career stages. Candid conversations with them help me remember that all of us have similar problems—sometimes daunting ones—but you’re not alone in facing them. Having someone else around to cheer for you when the science finally works is *well worth* the price of admission (i.e., time and reciprocity).

## Reality of the job—Be bold and take some risks

You were hired into academia to establish a scientific vision that might not yet exist. It was your previous track record that got you here, but you will now have to prove that you can stand on your own. The least perilous way to do that is by sampling from the types of work that you’ve already attempted. However, you *must* break out in new directions in which you might not have an ‘established’ track record in the conventional sense. This would be easy if every experiment you ever performed worked perfectly. But if science worked the first time, we would call it ‘search’ not ‘*re-*search’ (a sage proverb passed on by Gerry Fink to my wife when she was a graduate student). There is risk because you need simultaneously to manage scientific productivity with the *daring* to attempt feats never before attempted—some of which might fail.

In this complex balancing act, I learned two things about myself. First, that I was too cautious when setting up my lab. I continually felt like things were not quite ready—my ideas were not quite developed enough to write a grant proposal, my writing was not quite clear enough to submit a paper, and my funding did not feel quite secure enough to push hard on recruitment. Second, I concentrated thought on the structure of what I was doing—planning for the near horizon (i.e., six months into the future). I focused heavily on hitting all of the marks to make tenure, rather than really thinking ‘outside of the box’. Both phenotypes were the consequence of my attempts to manage risk, and I often came to the conclusion that I did not have time to fail *at all*. This was a trap I laid for myself.

In hindsight, I did make tenure, but I think I played it too safe. In the post-tenure career stage, I now feel the burn of my previous reticence. You can’t *always* play it safe, selecting the path that you know for sure will work out. It can help to talk with mentors about managing risks—by discussing perceived problems, an unexpected solution might surface. Although I believe that I played it too safe, I’m still not sure how one might fully avoid this trap.

## Job skill—Implement your vision—Through your people

It is crucial to recognize that the role you now occupy is different from your previous research position. You have been recruited to build a sustainable, independent research program. Your vision and audacity to pull it off landed you the job. You will need a great *team* to execute the experiments that will make the discoveries for which your lab will be known.

For me, it was quite helpful to have managed scientific work *through* research staff before I started my tenure track job. Look for opportunities at your institution to participate in training workshops for mentorship or leadership. If you find opportunities to mentor, use that time to learn what works for you and your mentees, realizing that each is unique in their interests, capabilities, and capacities for endurance. While this process can slow down the progress of projects in the short term, good management of your team yields exponentially greater benefits in the long run. Be open to adapting to people and projects, and always work to improve your mentorship approach. Your ability to help your trainees reach (even exceed) your level of understanding and execution is a *real trick* to success.

## Job skill—Become an efficient, masterful adjudicator

A less often mentioned skill that is essential to success in this job is how effectively you can judge quality. You will need to evaluate people—for example, determining whether prospective trainees have the right skills and mettle to do research; assessing a student’s performance on an exam or in a thesis committee meeting; listening to job and chalk talks to help select new faculty members for your department. You will need to evaluate the likely success of lab projects—when to double-down on an unexpected result; when to cut the project loose. You will be a peer-reviewer for manuscripts and grant proposals, deciding which projects are meritorious and worthy of advocacy. You will need to choose how to render criticism—how tough you decide to be, and on what areas you opt to focus your attentions and criticism. All of the adjudication built into the job can be *exhausting*, even all-consuming if you allow it. The practice of *quickly* making accurate and fair assessments of scientific quality is a time-saving crucial skill for your long-term success.

Start early on developing your skills as a critic. Find opportunities to review—read drafts of papers, offer feedback to your colleagues. Don’t be shy about making yourself available to scientific journals as a peer-reviewer, either jointly with an advisor, or on your own when you have accrued enough experience. Mentor others to hone your evaluation skills, not only on the science itself but also on how well your mentee understands and presents it. Create a lab environment that facilitates honest and respectful feedback and critique amongst team members. It has been extremely satisfying to watch my (now) senior graduate students give helpful feedback to the newest students in my group regarding their research and presentations.

## Job skill—Prepare for writing, writing, and more writing

Expect a substantial increase in both the amount and different types of writing tasks you will need to accomplish. It comes in the form of grant proposals, trying to convince others that your research merits funding. It comes in the form of scientific manuscripts, where you need to convince the *third* reviewer that your scientific inferences are correct. Eventually, you’ll be asked to write news-and-views, an altogether different style and messaging. Then, you’ll be asked to review grants—requiring yet another style and approach. You were always reviewing papers, but you’ll get asked to review more often and for increasingly important journals for your field. You’ll be asked to write locally and on the national stage. And that’s not counting all the tweet storms you’ll be typing when you get worked up, the requisite bits for your lab’s website, short bios for invited talks, recommendation letters, or your personal statement for your promotion dossier!

I regretted not being a more practiced writer when I started this job. Had I been, I think many things would have been easier, or at least more efficient. But why should *you* wait? Write now, write often, and hone this essential skill.

## Reality of the job—Self-assess and be pliable

Life as a professor requires multi-task management of your research, grant writing, service, teaching, lab management, not to mention your life outside of academia. Early on, I often felt like the progress that I made in one area (e.g., teaching or grant writing) often came with costs in other areas (e.g., research), owing to the intrinsic limits of time. Moreover, new opportunities will present themselves, and you’ll want to take full advantage. Simply sleeping less and working longer hours does not immediately solve these challenges!

What I found was that trying to balance all tasks meant two things. First, self-assessment at regular intervals of what I thought was going well, and what was not and thus required renewed focus. I needed to be honest and tough with myself about where things stood, but also be fair regarding tasks that seemed to be working. Once identified, I then needed mental discipline to shift my focus to whichever area required more attention, even if that meant refocusing on less-exciting tasks.

I also saw this to be true when evaluating trainees advancing in their careers. It turns out that they need to self-assess and sometimes refocus, as well. Being able to recognize what trainees need (even before they realize it) and steering them in the right direction is a second *real trick* that makes a mentor great.

## Job skill—The artful finesse of “no”

When you begin your tenure-track position, you will feel pressure to do whatever is asked of you. Mentors might advise you not to commit to everything that you are asked to do; yet these same advisors may turn right back around and ask for your service and time. I, personally, am intrinsically inclined to say “Sure!*”* to just about anything that’s asked of me, so adjusting this mindset was (and remains to this day) a challenge.

I slowly began to realize that I needed always to be asking myself the question: what do I *really* want to be working on? I see now that “yes” and “no” are tools of precision to be used strategically. Strategic use of these tools depends on many factors. Most importantly, does what you are being asked to do fit into the scope of your career and personal objectives? But somewhat less obvious is the importance of assessing the motivations of the people asking and what relationships you want to build with them. Some people are genuinely trying to help you. If those folks are asking you for something, you should trust them—listen and seriously consider a “yes” response. Other people simply want you to solve their problems—keep in mind that these are not your problems to solve. Learn to see the difference between these two types of requests. Make commitments that build supportive relationships and develop your broader career goals.

## Reality of the job—Be shrewd with your time—It is most valuable

Soon after minting your new independent lab, it might feel like you have *so much time* on your hands. That feeling will not last. You will rapidly take on many obligations. Your time will quickly become more limited than ever and the *value* of your time will be higher than ever. It follows that not everything that you might spend your time on is *actually really worth* your time. Of course, you will not be able to completely avoid all tedium. But you should enrich for time spent on work that is truly important (and enjoyable) and administrative tasks that support the objectives of your lab.

A less obvious, and somewhat uncomfortable, point about managing your time is that you also have to be mindful about time allotted to people and the scientific relationships that you have with them. There is ultimately a cost–benefit balance that one needs to monitor. Sometimes collaborations fail to thrive and you have to be willing to end them to preserve your time in the long run.

## And finally...

The good news is that, with the right motivation, it is very possible not just to succeed, but also to thrive in a tenure track academic position even if you aren’t fully prepared at the outset. Benjamin Franklin is attributed with saying “Motivation is when your dreams put on work clothes.” My experience is that, if you are motivated to succeed at the position, a lot of hard work is the pathway to promise. While the demands of a tenure track academic position are challenging without doubt, it is a rewarding position provided that you keep perspective about why *YOU* want the job (science, mentorship, teaching, etc.), and avoid the traps that make it unnecessarily more difficult.

## References

[1] Lappalainen T (2015). From trainee to tenure-track: ten tips. Genome Biol.

